# Correction to: Morbidity and mortality outcomes of COVID-19 patients with and without hypertension in Lagos, Nigeria: a retrospective cohort study

**DOI:** 10.1186/s41256-021-00215-1

**Published:** 2021-08-13

**Authors:** Akin Abayomi, Akin Osibogun, Oluchi Kanma-Okafor, Jide Idris, Abimbola Bowale, Ololade Wright, Bisola Adebayo, Mobolanle Balogun, Segun Ogboye, Remi Adeseun, Ismael Abdus-Salam, Bamidele Mutiu, Babatunde Saka, Dayo Lajide, Sam Yenyi, Rotimi Agbolagorite, Oluwatosin Onasanya, Eniola Erinosho, Joshua Obasanya, Olu Adejumo, Sunday Adesola, Yewande Oshodi, Iorhen E. Akase, Shina Ogunbiyi, Adenike Omosun, Femi Erinoso, Hussein Abdur-Razzaq, Nike Osa, Kingsley Akinroye

**Affiliations:** 1 Lagos State Ministry of Health/Lagos Incident Management Command System, Lagos, Nigeria; 2 College of Medicine, University of Lagos, Idi-Araba, Lagos, Nigeria; 3 Lagos State Primary Health Care Board, Lagos, Nigeria; 4 Mainland Hospital, Yaba, Lagos, Nigeria; 5 Lagos State University College of Medicine, Lagos, Nigeria; 6 Lagos State Biobank, Lagos, Nigeria; 7 World Health Organization, Nigeria Office, Abuja, Nigeria; 8 Nigeria Centre for Disease Control, Abuja, Nigeria; 9 Lagos University Teaching Hospital, Lagos, Nigeria; 10 Lagos State University Teaching Hospital, Lagos, Nigeria; 11 Nigerian Heart Foundation, Lagos, Nigeria

Correction to: Global Health Research and Policy (2021) 6:26 https://doi.org/10.1186/s41256-021-00210-6

Following publication of the original article [1], it is reported the second paragraph of the introduction includes some errors, as is indicated below.

The original first sentence was:

Much earlier in a study in China, where COVID-19 was initially identified, it was found that 48% of patients had a comorbidity, with hypertension being the most prevalent (30%), followed by diabetes (19%) and coronary heart disease (8%) [7].

The correct first sentence should be:

Much earlier in a study in China, where the COVID-19 was first reported, it was found that 48% of patients had a comorbidity, with hypertension being the most prevalent (30%), followed by diabetes (19%) and coronary heart disease (8%) [7].

The original article [1] has been updated.
